# Correction: Loss of a Conserved tRNA Anticodon Modification Perturbs Plant Immunity

**DOI:** 10.1371/journal.pgen.1005911

**Published:** 2016-02-26

**Authors:** Vicente Ramírez, Beatriz Gonzalez, Ana López, María José Castelló, María José Gil, Graham J Etherington, Bo Zheng, Peng Chen, Pablo Vera

Graham J Etherington should be included in the author byline. He should be listed as the sixth author and his contribution is: Genome sequencing and SNPs identification in the mutant plant. His affiliations are 1: The Sainsbury Laboratory, Norwich Research Park, Norwich, United, Kingdom, and 2: The Genome Analysis Centre, Norwich Research Park, Norwich, United Kingdom. The correct citation is: Ramírez V, Gonzalez B, López A, Castelló MJ, Gil MJ, Etherington GL, et al. (2015) Loss of a Conserved tRNA Anticodon Modification Perturbs Plant Immunity. PLoS Genet 11(10): e1005586. doi: 10.1371/journal.pgen.1005586
